# Ischaemic heart disease and pregnancy: the tale of two stories

**DOI:** 10.5830/CVJA-2017-050

**Published:** 2018

**Authors:** R Matshela Mamotabo

**Affiliations:** University of KwaZulu–Natal, Durban; Mediclinic Heart Hospital, Pretoria, South Africa; London School of Economics and Political Science, London, UK

**Keywords:** pregnancy, ischaemic heart disease

## Abstract

Ischaemic heart disease (IHD) is presumed to be rare in pregnancy. Based on that assumption, patients go undiagnosed or undertreated. IHD in pregnancy frequently occurs as a result of an unusual aetiology, therefore each patient needs to be managed individually since each may present differently. This may pose challenges to the consulting clinician. Pregnancy itself is a risk factor for cardiovascular disease, due to its associated hypercoagulable state. From current reports, the prevalence of IHD in females is increasing due to lifestyle changes, including cigarette smoking, diabetes and stress. In our modern societies, women delay childbearing until they are older, allowing time for risk factors to cluster. Although presumed to be rare in pregnant women, IHD is currently estimated to occur three to four times more often during pregnancy in middle– and high–income women, warranting an extensive review highlighting cases of IHD in pregnancy.

In the developing world, hypertension and rheumatic disease are the commonest heart diseases encountered in pregnancy. However, the prevalence of adult congenital heart disease and other cardiovascular diseases is increasing. Pregnancy itself is a risk factor for cardiovascular disease due to its associated hypercoagulable state.^1^,^2^

The era of human immunodeficiency virus and improved socio–economic lifestyles has ushered in a rise in the frequency of ischaemic heart disease (IHD) in pregnancy. However, many patients go undiagnosed and undertreated due to the assumption that IHD is rare in pregnancy. IHD, particularly acute myocardial infarction in pregnancy, frequently occurs due to an unusual aetiology and as a result, each patient needs to be managed accordingly.

Cardiac disease in pregnancy remains a minor yet significant cause of maternal mortality worldwide. Since rheumatic fever is uncommon in affluent societies, most cases in the Western world are the result of congenital heart disease. In the developing world, rheumatic heart disease remains the major cause of cardiac disease in pregnancy.^3^–^7^ The National Committee on Confidential Enquiries into Maternal Deaths lists cardiac disease as one of the five major causes of maternal death in southern Africa.^8^,^9^

Until recently, IHD or coronary artery disease (CAD) in pregnancy has been described as a rare occurrence. In southern Africa, there have been a few reports on IHD, including acute myocardial infarction (AMI)/acute coronary syndrome (ACS) in pregnancy. The purpose of this article is to describe the challenges one encounters regarding clinical presentation and management of IHD in pregnancy, particularly AMI. We shall also highlight each patient’s clinical presentation and some challenges encountered by cardiologists and obstetricians to provide appropriate care.

## Case_reports

**Fig. 1:**
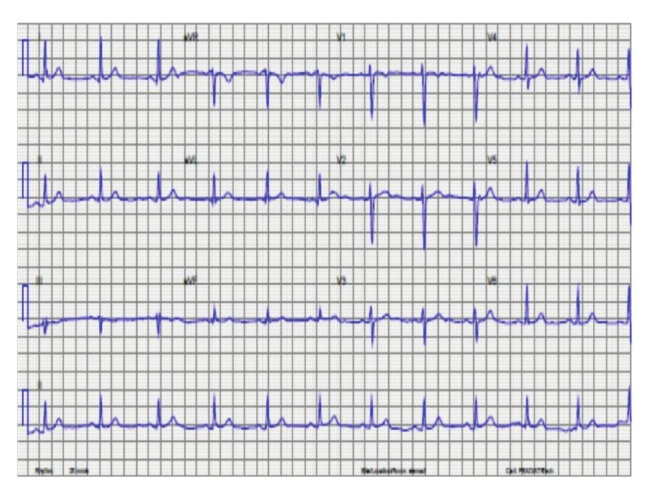
Electrocardiogram of patient 1 in sinus rhythm showing an isolated T–wave inversion and poor R amplitude in lead III.

Patient 1 was a 42–year–old white female primigravid who presented at 33 weeks of gestation complaining of Canadian Society class (CCS) II angina and New York Heart Association (NYHA) grade II dyspnoea. In addition, she had a past history of an inferior wall ST–segment elevation myocardial infarction six months prior to her pregnancy and she was also epileptic. Her additional risk factors for CAD included type 1 diabetes mellitus and hypertension. The clinical examination was unremarkable and her initial transthoracic electrocardiogram revealed sinus rhythm with poor R–wave amplitude in lead III and T–wave inversion in leads III and V1 (Fig. 1).

A transthoracic echocardiogram revealed preserved left ventricular (LV) function with an ejection fraction of 58%, and basal septal wall hypokinesia. An emergency caesarean section was performed during her admission due to worsening symptoms and recurrent episodes of seizures, and foetal distress. The peri–operative period was uneventful and she delivered a 1.8–kg female baby with dimorphism, in keeping with trisomy 21.

Patient 2 was a 34–year–old female of Indian descent, gravida 2 and para 1, who presented at 20 weeks of gestation, with a past history of an extensive anterior and inferior myocardial infarction at the age of 25 years. The patient had suffered a spontaneous dissection of the mid–left anterior descending (LAD) artery during her first pregnancy, which was confirmed by an intravascular ultrasound. Her symptoms during her current pregnancy included CCS class II angina and NYHA grade III dyspnoea. In addition, she had a past history of hypercholesterolaemia.

**Fig. 2:**
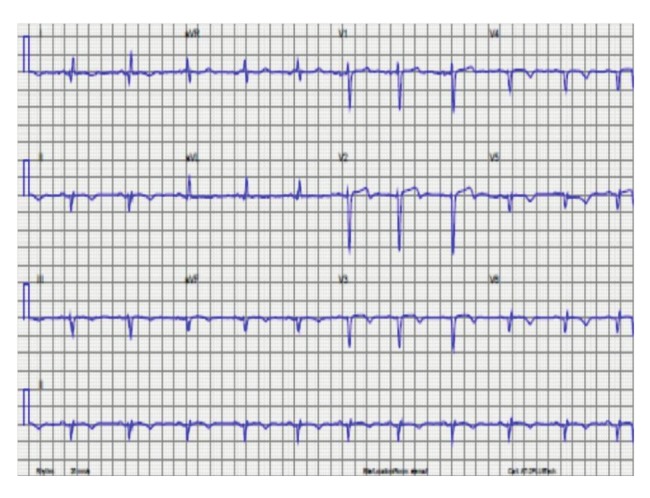
Electrocardiogram of patient 2 showing sinus rhythm, poor R amplitude and T–wave inversions globally, with a left–axis deviation.

Her initial electrocardiogram revealed poor R amplitude and T–wave inversion globally, and Q waves in the inferolateral leads (Fig. 2). A transthoracic echocardiogram revealed a dilated left ventricle and impaired LV function with an ejection fraction of 38%, and multiple regional hypokinesia with no evidence of LV mural thrombus. A repeat coronary angiogram performed after delivery revealed normal epicardial coronary arteries with severely impaired LV contractility, with an estimated LV ejection fraction of 35% and a large LV apical (mural) thrombus. The patient delivered a 3.09 kg healthy female baby by an elective caesarean section after 34 weeks of pregnancy. There were no intra–operative or postoperative complications reported.

With subsequent follow up, her heart failure had worsened and a repeat transthoracic echocardiography revealed deterioration in her LV function, due to poor compliance. Her anti–failure therapy was optimised and her condition improved dramatically, however she was lost to follow up.

Patient 3 was a 34–year–old female of Indian descent who presented to her local hospital at 33 weeks of gestation with an acute anterior ST–elevation myocardial infarction. However, the patient was referred to the tertiary hospital at least 24 hours after her initial presentation to her local hospital. In addition, there was a documented history of an acute myocardial infarction a year earlier. Her risk factors for CAD included diabetes mellitus and hypertension.

**Fig. 3:**
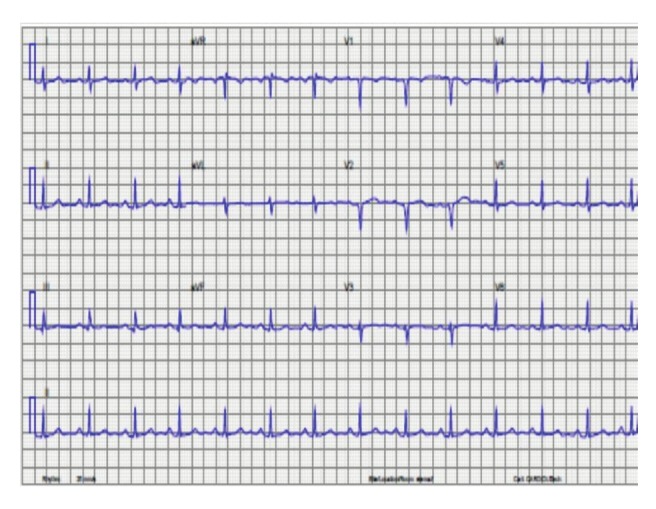
Electrocardiogram of patient 3 at least 24 hours after the myocardial infarction, in sinus rhythm with Q waves in leads V1–V2 (poor R–wave amplitude in leads V1–V3).

**Fig. 4:**
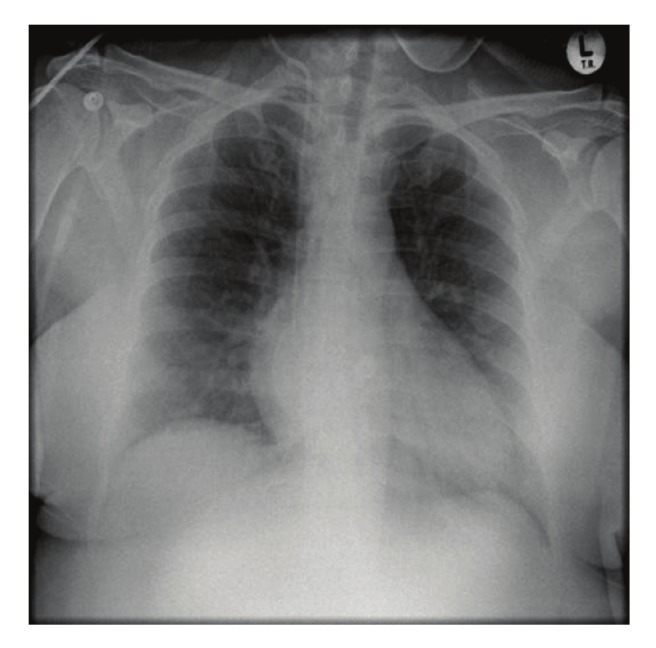
Chest X–ray of patient 3 showing a mildly increased cardiothoracic ratio and clear lung fields.

Her initial electrocardiogram revealed extensive ST elevations in the anterior leads. A repeat electrocardiogram on arrival at our tertiary hospital, which was at least 24 hours later, revealed sinus rhythm with evidence of a recent extensive antero–septal myocardial infarction (Fig. 3). Her chest X–ray is shown in Fig. 4.

**Fig. 5:**
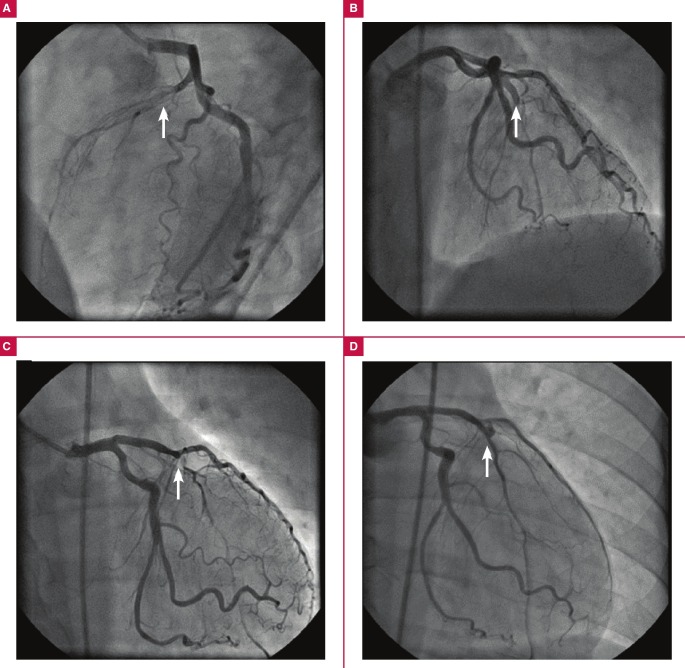
Patient 3. (A–C) Coronary angiogram showing the sub–totally occluded left anterior descending coronary artery (LAD, indicated by the white arrows). (D) Improved flow post–PCI and stenting to the proximal LAD with TIMI 3 flow to the distal vessel (white arrow).

Echocardiography revealed preserved LV function with an ejection fraction of 62% and regional wall motion abnormalities, including antero–septal and infero–basal hypokinesia. Coronary angiography revealed a sub–totally occluded LAD (Fig. 5).

Percutaneous revascularisation was performed where a baremetal stent was successfully deployed with no peri–procedural complications (Fig. 5). Strict radiation protective measures of the foetus were undertaken during the procedure.

An elective caesarean section was performed two weeks later, when a 2.2–kg female baby was delivered with no peri–operative complications. During her cardiology follow up, she reported severe prolonged rest pain, which was attributed to poor compliance. Her management was optimised and her symptoms improved.

## Discussion

**Acute myocardial infarction**

In the largest series of AMI in pregnancy where 125 patients were reported, the authors discovered the highest incidence of AMI in the third trimester, and most importantly, in multigravid women older than 33 years of age.^1^,^2^,^10^–^12^ In the same report, a coronary angiography was performed in 54% of the patients; 43% of these had coronary atherosclerosis and 21% had coronary thrombus without evidence of atherosclerotic disease. In addition, 16% had spontaneous coronary artery dissection, while 29% had normal epicardial coronaries.^10^,^11^ Anterior wall myocardial infarction was the most common occurrence to be reported.

In earlier reports, myocardial infarction was associated with a maternal mortality rate of 21% or higher. However in recent reports, the mortality rate was estimated to be between 5 and 11%, and most foetal deaths were associated with maternal deaths.^1^,^2^,^10^–^12^ Most importantly, most maternal deaths occurred at the time of an acute myocardial infarct or within two weeks of the acute event, usually related to labour and delivery.

A literature survey by Ladner et al.^13^ reported a total of 151 women with AMI, yielding an incidence rate of one in 35 700 deliveries. This report was supportive of the study by Roth et al.,^10^,^11^ who reported that AMI was more common in women older than 35 years and often in multiparous women. Important independent risk factors for AMI included chronic hypertension, diabetes, advanced maternal age, eclampsia and severe pre–eclampsia. The same authors reported a maternal mortality rate of 7.3% and also indicated that maternal death only occurred in those women with AMI before or at the time of delivery.^10^,^11^ In addition, the authors reported that 38, 21 and 41% of the incidences occurred during the antenatal and intrapartum periods and six weeks postpartum, respectively; and the incidence of AMI increased over the 10–year study period.^1^,^2^,^14^

Hankins et al.^12^ emphasised that delivery within two weeks of infarction was associated with increased mortality rates and in addition, an increased risk of re–infarction occured during labour. The reporters indicated that increased cardiovascular stresses late in pregnancy, especially when intensified by parturition, compromise women with IHD. As a result, efforts should be made to limit myocardial oxygen demand/consumption throughout pregnancy and particularly during parturition.

**Percutanous coronary intervention (PCI) during pregnancy**

James et al.^1^,^2^ reported on revascularisation by PCI in a total of 135 patients, with stent deployment in 127 of the patients.^1^,^2^ However, information on outcomes was limited.

In another study, more data were reported on 92 of 103 patients who had coronary angiography, where 49 and 43 of these patients had the procedure during the antepartum and postpartum periods, respectively.^15^–^17^ PCI was subsequently performed in 38 (41%) of the patients (23 antepartum, six peripartum and nine postpartum) with stent placement in only 55% of the patients and bare–metal stents deployed in all patients. Drug–eluting stents should be avoided during pregnancy if possible due to limited information on their safety.^15^–^17^

**Coronary arterial bypass grafting during pregnancy**

Although information regarding the safety of coronary arterial bypass grafting (CABG) during pregnancy is limited, James et al.^1^,^2^ reported surgical revascularisation in 61 women with AMI during pregnancy. Despite this impressive number of patients who had undergone surgical revascularisation, information on the outcome of these procedures was not provided.

Dufour et al.^18^ reported data on pregnancy in patients who had had a previous myocardial infarction with or without prior CABG. In the majority of patients who died, their death occurred at the time of myocardial infarction, and maternal mortality rate was the greatest if myocardial infarction occurred late in pregnancy.

**Prevalence of acute coronary syndromes (ACS) during pregnancy**

The prevalence of myocardial infarction during pregnancy was previously estimated at one per 10 000 pregnancies, however current estimates indicate that the prevalence has increased three to four times, more often in middle– and high–income women.^19^–^25^ Myocardial ischaemia during pregnancy can mimic typical symptoms related to pregnancy itself, which may be misinterpreted, resulting in under–reporting of the incidence. When myocardial infarction does occur, it may be associated with both maternal and neonatal mortality, and the risk increases during the peripartum period, particularly during labour and within a few weeks of delivery.

Although most cases of myocardial infarction in non–pregnant patients are due to coronary atherosclerosis, alternative aetiologies should always be looked for in pregnancy. Pregnancy is a hypercoagulable state, which increases the risk of AMI, as does older age at the time of conception.^23^–^25^ Changes in the cardiac, haemodynamic, haemostatic and hormonal milieu during pregnancy and the puerperium period create a spectrum of stresses that may provoke ACS. Spontaneous coronary dissection is one of the commoner and more important causes of ACS in these patients.^15^,^26^–^41^

## Conclusion

Although IHD was previously presumed to be rare in pregnancy, current reports estimate a three– to four–fold increase, more often in middle– and high–income women. Pregnancy, due to its associated hypercoagulable state, is a major risk factor for cardiovascular disease in the current era. Changes in lifestyle, including cigarette smoking, diabetes and stress, and delayed childbearing until older age further increase the risk of IHD in pregnancy.

The management of IHD in pregnancy, particularly AMI, remains controversial due to limited data. Bare–metal stents are reported to be the preferred choice of intervention compared with drug–eluting stents due to limited information and risk of prolonged anticoagulation. In addition, since information regarding the safety of CABG during pregnancy is rather limited, CABG should not be recommended as the first option.
